# Decoding the Microbial Diversity of Indian Fermented Foods: Integrating Ethnobiology, Multi-Omics and Functional Insights

**DOI:** 10.3390/foods15040687

**Published:** 2026-02-13

**Authors:** Priyanka Samantaray, Sudeshna Saha

**Affiliations:** Department of Biological Sciences, SRM University AP, Amaravati 522 240, Andhra Pradesh, India; priyanka_samantaray@srmap.edu.in

**Keywords:** fermented foods, glycemic value, omics, microbiome, India

## Abstract

India’s diverse culinary heritage includes a wide spectrum of traditional fermented foods that harbour complex microbial communities essential for flavour development, preservation, and nutritional enhancement. These microorganisms—primarily lactic acid bacteria, yeasts, and molds—contribute functional properties that extend beyond food transformation to confer health benefits, including probiotic potential and metabolic regulation. This review integrates classical microbiological studies with modern molecular approaches such as metagenomics, metatranscriptomics, and metabolomics to elucidate the microbial diversity of Indian fermented foods. It highlights how geography, substrates, and ethnic traditions shape region-specific microbial consortia sustained through long-standing ethno-microbiological practices. Special focus is given to the glycemic modulation achieved through microbial fermentation, wherein organic acid production and resistant starch formation lower glycemic index and improve glucose metabolism. These processes, along with enhanced nutrient bioavailability, vitamin synthesis, and immunomodulation, illustrate the broader functional potential of fermentation. The review also examines interactions between food-borne microbes and the human gut microbiota, underscoring implications for personalized nutrition. Finally, it discusses modernization and commercialization strategies and outlines future directions involving multi-omics integration, indigenous starter cultures, and microbiome-based innovations to harness India’s microbial heritage for improved health and sustainable food development.

## 1. Introduction

India’s culinary heritage, shaped over millennia by geographical diversity, climatic variations, and cultural plurality, encompasses a vast array of fermented foods that reflect the interconnection between biodiversity, traditional knowledge, and nutrition. Fermentation, a biotransformation process driven by diverse microbial communities, plays a pivotal role in improving the nutritional value, sensory profile, shelf life, and functional properties of foods. These natural microbial consortia, comprising bacteria, yeasts, and fungi, carry out complex biochemical conversions that enhance bioavailability of nutrients, generate bioactive compounds, and potential health benefits.

Over the past few decades, advances in culture-independent and high-throughput molecular technologies have increased the exploration of microbial consortia associated with traditional Indian fermented foods. By integrating conventional culture-based methods with multi-omics tools such as metagenomics, metaproteomics, and metabolomics, researchers have begun to decode the ecological and functional complexity underlying these foods. This paper provides a comprehensive review of the microbial diversity inhabiting India’s fermented foods, highlights their biochemical and nutritional characteristics as well as regional specificity, and finally presents Indian fermented foods in the context of today’s global food market.

## 2. Historical Perspective of Fermented Food in India

Ancient Indian fermentation practices trace back over 3000 years and were deeply intertwined with agriculture, religion, medicine, and collective tribal life. Archaeological and textual sources reveal that fermentation was not only a technological process but also a spiritual and subsistence cornerstone of Indian civilization. Vedic literature such as the Rigveda described early fermented beverages like Soma and Sura, prepared using grains, herbs, and ferments, which marked the beginning of systematic fermentation in India [1,2]. These methods evolved with settled agriculture during the Neolithic period, where grains and fruits were spontaneously fermented by undefined microbes. Texts like Charaka Samhita and Sushruta Samhita discussed the medicinal uses of fermented preparations (Asava, Arista, Amla), suggesting early biochemical knowledge of microbial transformations. Subsequently, Kautilya’s Arthashastra mentioned vinegar (Amla) production and fruit-based fermentations used both for flavouring and preservation. Fermented foods and beverages held ritual significance in Vedic and later Hindu traditions. Soma was considered sacred, symbolizing divine vitality and immortality. In many regions, fermented milk products such as buttermilk (takra) and curd (dahi) were used in religious rituals and offerings. In Ayurveda, fermented drinks like Kanji, Peya, and Takra were recommended for seasonal balance and digestive health, reflecting how spiritual, ecological, physiological and nutritional understandings were integrated into everyday dietary practices [3]. Hindu dietary treatises also categorized fermented products under specific therapeutic or ritual contexts, acknowledging both their vitality and their constrained use during purification rites.

Furthermore, fermentation was essential for food preservation and survival in pre-refrigeration agrarian societies. It transformed perishable surpluses from harvests into long-lasting products such as kanjika (fermented cereal gruel), sukta (vinegar-like condiment), and pickles used throughout the year. In rain-fed and tribal agricultural systems, fermentation ensured nutritional security, enhanced digestibility, and promoted probiotic health benefits. The technology required minimal energy, making it sustainable for rural communities. Across India’s tribal belts, from the Bodo and Naga communities of Northeast to Gond and Bhil tribes in central India, fermentation shaped local food identities and social cohesion. Rice beers like Apong, Zutho, and Judima were central to festivals and community rituals that celebrated harvests and kinship. Fermented fish, soybeans, and bamboo shoots served as protein-rich conservation foods in hilly terrains with limited meat availability. These traditions persist today, maintaining deep ecological links between indigenous microbiota, agro-biodiversity, and ethnic heritage. Overall, ancient Indian fermentation was a holistic system combining empirical microbiology, sacred symbolism, and ecological mindfulness—providing a foundation for the country’s enduring microbial diversity in food cultures.

## 3. Molecular Technologies Advancing Understanding of Indian Fermented Foods

**Figure 1:**
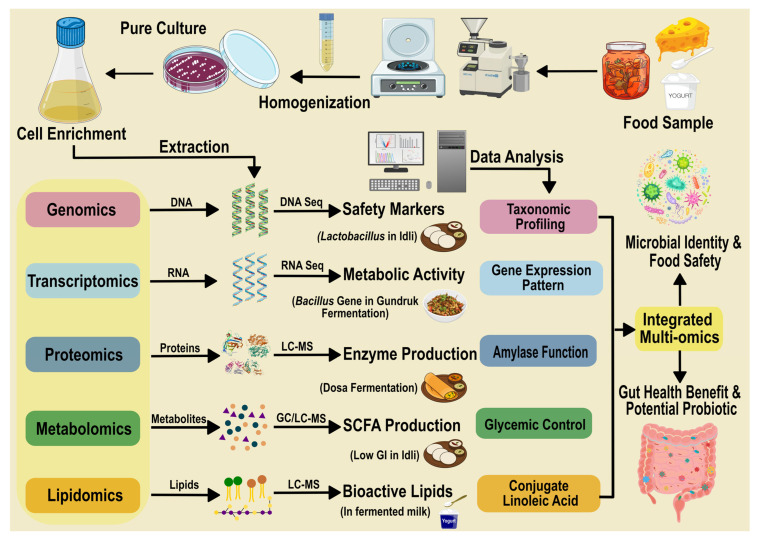
Schematic showing the integration of various omics techniques which is used to determine the functional and probiotic assessment of fermented foods. Each of the technique rely on the extraction of specific biomolecule from the microbial enrichments of the food and can be analyzed to identify characteristic functional properties associated with the food microbiome.

Industrial advancement has accelerated the commercial development and global recognition of Indian fermented foods. Traditional products like dahi, lassi, and fermented snacks are now produced commercially, aligning with the global shift toward functional and probiotic-enriched foods. Parallels may be drawn with Asian fermented foods such as miso (Japan) and kimchi (Korea), yet India’s substrate variety, ethnocultural foundations, and microbial diversity render its fermented foods uniquely multifaceted [4]. The understanding of the microbial repertoire of Indian fermented foods has benefitted through the development of modern technological advancements. Recent advances in molecular biology techniques have revolutionized the study of microbial communities in Indian fermented foods by offering detailed, high-resolution insights into their diversity, functions, and dynamics throughout fermentation (Figure 1).

- 16S rRNA gene sequencing has helped identify the wide range of bacterial species, including dominant and rare taxa, involved in fermentations like idli and dosa. Although it fails to provide functional insights of the food microbiome, this technique has provided foundational taxonomic profiles that were previously inaccessible by culture-based methods; thus, enabling a more complete understanding of microbial community composition and its regional variability [5].
- Metagenomics has expanded this understanding by characterizing entire microbial consortia, including bacteria, fungi, and unculturable microbes. It has uncovered functional genes responsible for fermentation pathways, nutritional enhancement, and probiotic traits, thus linking microbial presence with potential health benefits and fermentation efficacy [6].
- Low resolution DNA fingerprinting methods like DGGE (Denaturing Gradient Gel Electrophoresis) and TGGE (Temperature Gradient Gel Electrophoresis) have allowed researchers to assess microbial succession and diversity changes during fermentation processes. These methods have helped revealing how microbial communities evolve over time and how different environmental or processing factors impact fermentation quality and safety [7].
- Quantitative PCR (qPCR) enables precise measurement of key microbial groups and functional genes, permitting real-time monitoring of fermentation stages and microbial kinetics. This has improved control over fermentation consistency and product safety while enhancing understanding of important microbial growth trajectories [8,9].
- Next-generation sequencing (NGS) offers strain-level resolution and deep sequencing depth that reveal intra-species diversity and genetic variations influencing product characteristics and health effects. This level of detail supports tailoring fermentation protocols for desired sensory or nutritional outcomes, as seen in detailed studies of idli batter microbiota [10].
- Metatranscriptomics and metaproteomics have uncovered which microbial genes and proteins that are actively expressed during fermentation, offering functional insights into metabolic pathways, biosynthesis of flavour compounds, and bioactive substances. This dynamic analysis deepens understanding of microbial roles beyond mere presence and help provide an unbiased biochemical profiling of the microbial flora associated with the food [11].
- Fluorescence in situ hybridization (FISH) combined with multi-omics approaches provides spatial localization of microbes within the food matrix and integrates genomic, transcriptomic, proteomic, and metabolomic data to elucidate microbial ecosystem structure and function. This holistic view enables comprehensive insight into microbial interactions essential to traditional Indian fermented food complexity [12].

These molecular and multi-omics technologies ranging from 16S rRNA sequencing and DGGE/TGGE for taxonomic profiling and succession tracking, to qPCR for targeted quantification, NGS and metagenomics for strain-level and functional gene insights, metatranscriptomics/metaproteomics for active pathway elucidation, and FISH/multi-omics for spatial organization, have collectively revolutionized the understanding of Indian fermented foods like idli and dosa. Collectively, these molecular tools have revealed the vast microbial biodiversity, metabolic potential, and health-related bioactive compounds (such as bioactive peptides) inherent in Indian fermented foods. Previously opaque “black box” processes are now characterized as dynamic microbial ecosystems with conserved consortia of lactic acid bacteria and yeasts driving acidification, leavening, flavor, nutrition, and safety, while revealing regional variability and key interactions. They have enabled precise monitoring and optimization of fermentation processes while linking traditional knowledge with modern science. These insights support innovation in food safety, nutritional enhancement, personalized nutrition, and functional food product development, ensuring that Indian traditional fermentation practices can be scientifically validated, preserved, and technologically advanced to meet modern demands [13].

## 4. Classification of Indian Fermented Foods and Microbial Habitats

India’s fermentation practices reflect ecological diversity, crop availability, and cultural plurality. Techniques such as spontaneous souring, backslopping, wrapped ripening, and amylolytic starter inoculation transformed milk, cereals, legumes, vegetables, fish, and flowers into functional foods embedded in daily diets, seasonal cycles, and rituals. Indian fermented foods are categorized based on substrate types, with distinct microbial profiles and functionalities associated with each category, as summarized in Table 1.

This categorization highlights the diversity of substrates and microbial communities shaping the properties of Indian fermented foods, deeply rooted in regional and ethnic traditions.

## 5. Microbial Constituents: Impact of Climate, Topography, Agriculture, and Substrate Choices

**Figure 2:**
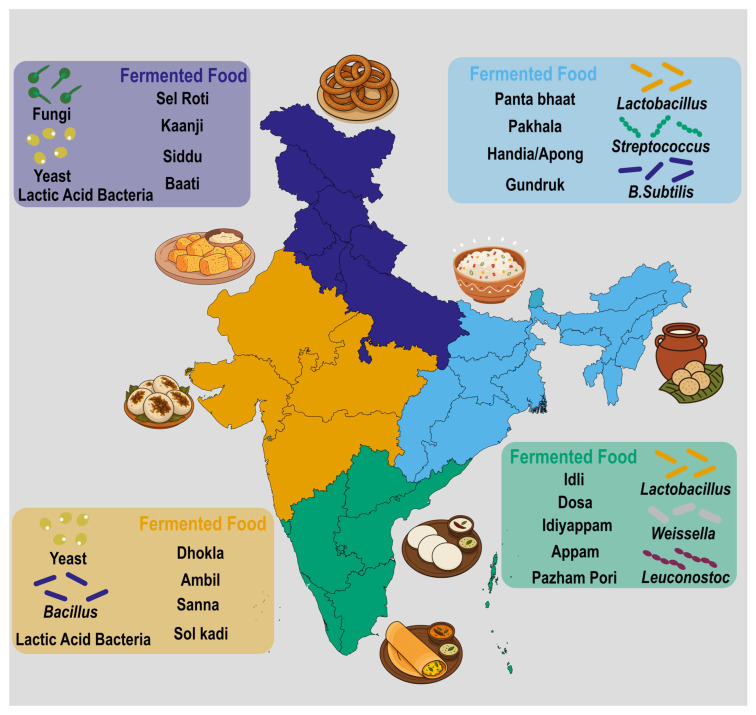
Geographical distribution and microbial diversity of Indian fermented foods. The microbial diversity of the fermented food is modulated by multiple factors. Environmental factors such as temperature, humidity, climate, altitude, and seasonal shifts select for resilient microbial strains adapted to local niches, while substrate chemistry (e.g., carbohydrate profiles, pH) and processing parameters like fermentation duration, salt/moisture levels, container materials, backslopping, and indigenous starters drive microbial succession and consortium stability. The primary regional distributions are indicated by distinct colors in the country map. The colored inset boxes represent the key fermented foods of the region and their microorganisms.

India’s fermented foods evolved through long co-evolution between cultural practice and microbial ecology across diverse substrates, such as dairy, cereals and millets, legumes, vegetables, bamboo shoots, fish, and plant saps and heterogeneous processing environments influenced by climate and product culture [15,16,17] (Figure 2). Core microbial groups include LAB; yeasts including *Saccharomyces* and non-*Saccharomyces* species; and molds such as *Aspergillus* and *Rhizopus*. Community structure is shaped by substrate chemistry, temperature, salt and moisture, materials of the containers/equipment, and artisanal practices like backslopping and use of indigenous starters [18,19]. These microbial consortia drive specific functional outputs like rapid acidification for safety, proteolysis and amylolysis for texture and digestibility, production of organic acids and exopolysaccharides for glycemic modulation and mouthfeel, and synthesis of vitamins and bioactive peptides that underpin probiotic and immunomodulatory effects.

- Northern India (Indo-Gangetic Plain and Himalayan Foothills)

The agroecological landscape of Northern India, encompassing the Indo-Gangetic plain and Himalayan foothills, is defined by fertile alluvial soils, abundant freshwater availability, hot summers, and cool winters that support mixed rainfed and irrigated agriculture alongside a strong bovine dairy economy. This ecological richness has fostered diverse fermentation traditions, especially those based on dairy, cereals, legumes, and vegetables. Common fermented products include dahi, lassi, and mattha from milk; bhatura and papad from wheat and legumes; and kanji and brined pickles derived from vegetables such as black carrots. The microbial ecology of these foods is predominantly lactic acid bacteria (LAB) driven, featuring lactobacilli, *Lactococcus*, and *Leuconostoc* species that contribute to acidification, texture, and microbial safety. Fermentations are typically spontaneous or maintained through backslopping [17,19]. Brined vegetables, on the other hand, favour salt- and acid-tolerant LAB and osmophilic yeasts, with spice-derived phytochemicals influencing microbial succession and flavour development [20,21]. The prominence of these substrates in the region stems from extensive cattle and buffalo husbandry, favourable winter temperatures for lactic fermentations, and readily available wheat and legumes that facilitated the evolution of dough- and papad-based ferments [15,16].

- Western India (Semi-Arid Plateaus and Konkan Coast)

Western India exhibits high ecological contrasts between its semi-arid interiors, spanning Rajasthan and Gujarat, and the humid Konkan coast extending through Maharashtra and Goa. The interior regions are characterized by millet and pulse agriculture, while the coastal areas are dominated by rice, coconut, and fisheries. The interplay between these ecologies has shaped a vibrant fermentation repertoire. Staple products include dhokla, khaman, handvo, and thepla, derived from cereal/pulse batters; shrikhand and basundi from dairy; and chaas, a lightly fermented buttermilk beverage. Coastal zones additionally produce diverse brined and sun-fermented pickles influenced by high humidity and salinity. Microbially, rice–pulse batters nurture LAB and yeast consortia whose amylolytic and proteolytic activities create the desired leavening and sourness [17,18]. Coastal fermentations, in contrast, favour halotolerant LAB and yeasts, with exposure to the sun accelerating acidogenesis and enhancing food safety [16,21]. Millet and pulse resilience under drought conditions and coastal access to salt and marine resources partially indicate the region’s reliance on fermented batters, brined vegetables, and oil-preserved [16].

- Eastern India (Humid Deltas and Floodplains)

Eastern India’s humid, riverine environments, including the deltas of Bengal, the floodplains of Assam, and the coastal plains of Odisha, are characterized by high rainfall, fertile soils, and bamboo abundance. These features underpin a fermentation landscape centered on rice, dairy, and plant-based substrates. Iconic examples include pakhala, panta, and poita bhaat (soaked or fermented rice), mishti doi and chhena-based sweets, fermented bamboo shoots, and traditional rice beers such as handia and haria. Rice-based ferments harbour LAB and saccharolytic yeasts that produce mildly acidic and lightly alcoholic matrices, improving digestibility and shelf life [15,16]. Bamboo shoot fermentations, meanwhile, are LAB-dominant and influenced by endogenous plant enzymes, which shape the umami and aroma profile; microbial diversity varies with the use of local leaf wraps and vessel ecology [22]. The abundance of paddy, high humidity, and cultural practices of reusing cooked rice favoured the rise in rice fermentations, while bamboo-rich landscapes and foraging traditions sustained bamboo-based ferments [16,22].

- Southern India (Humid Tropics and Western Ghats)

Southern India’s humid tropical climate, influenced by the Western Ghats and monsoon rainfall, supports rice, millet, and pulse cultivation alongside extensive coconut and spice belts. These ecological conditions have produced one of India’s most iconic fermentation traditions: the cereal–pulse batter used in idli, dosa, uttapam, appam, and vada. Complementary dairy ferments such as curd rice and moru (spiced buttermilk), and beverages like toddy from coconut or palm sap, further enrich the regional diversity. The microbial ecology of batters features stable LAB, yeast consortia that acidify and leaven the mixture, while exopolysaccharide production enhances texture; fermentation kinetics are strongly temperature-dependent [17,18]. Toddy fermentations rely on sugar-fermenting yeasts cohabiting with LAB, progressing rapidly under warm, humid conditions [16,23]. The prevalence of these substrates is linked to the dominance of rice–pulse agriculture, vegetarian dietary traditions, and the natural availability of coconut and palm resources, which together fostered both food and beverage fermentations [15,24].

- Northeastern India (Hilly, High-Rainfall, Biodiverse)

The Northeastern region, encompassing states like Assam, Nagaland, and Manipur, is distinguished by steep terrain, heavy rainfall, and extraordinary biodiversity. Shifting cultivation systems, bamboo forests, and abundant fish resources have shaped a unique fermentation ecology. Major substrates include soybean (as in kinema, axone, hawaijar), leafy vegetables (such as gundruk, sinki, khalpi), and fish and meat products (ngari, tungtap). Rice-based fermented beverages like apong, zutho, and chhang, often initiated with household starters (marcha, hamei, ranu), are central to community life. The microbial ecology of these systems reveals proteolytic LAB and *Bacillus* species dominating soy and fish ferments, while vegetable ferments remain LAB-driven. Mixed LAB, yeast populations in rice starters perform saccharification and alcohol production [13,16,22]. Forest abundance, bamboo and soy availability, and dispersed household fermentation practices supported the emergence of robust microbial terroirs and resilient starter cultures across this region [16,22].

- Central India (Tribal Forest Belts and Plateaus)

Central India’s forested plateaus and tribal regions are characterized by small-scale millet and rice farming, non-timber forest products, and periodic water scarcity. Fermentation here is deeply tied to community traditions and ritual beverages. Key examples include handia, a fermented rice beer, and floral liquors derived from mahua (*Madhuca longifolia*). The microbial ecology of these beverages features amylolytic molds and yeasts that initiate saccharification, accompanied by LAB that stabilize the ferment [16,25]. Floral substrates contribute unique yeast and LAB profiles, creating regionally distinct flavours. These practices thrived due to the reliance on forest resources, the social role of fermented beverages, and the adaptability of household starter cultures to intermittent agricultural cycles [16].

- Himalayan and Hilly States (Alpine and Mid-Hill Ecologies)

In the Himalayan and mid-hill regions, encompassing parts of Uttarakhand, Himachal Pradesh, and Sikkim, cold winters and cool summers define an agroecology dominated by terraced cultivation of barley, buckwheat, and millets, alongside yak and cow husbandry. Fermentation here serves a vital role in food preservation and energy enhancement. Characteristic products include selroti, a sweet fermented rice bread; chhang and ara, barley-based beverages; chhurpi, a hard cheese from yak or cow milk; and gundruk, fermented leafy greens. The low ambient temperatures select for psychrotrophic LAB and yeasts, slowing fermentation and permitting extended ripening, especially in dairy products [19,26]. Leafy green fermentations proceed through salt-assisted lactic acid fermentation under anaerobic conditions. The regional emphasis on fermentation arises from the need for preservation in cold climates, the demand for energy-dense foods at high altitudes, and dependence on livestock in environments with limited crop diversity [15,16].

## 6. Indian Fermented Foods: Influence on Glycemic Content and Probiotic Potentials

Microorganisms in Indian foods contribute to health through probiotic traits, vitamin synthesis, and fermentation quality. *Weissella* and lactobacilli produce bioactive compounds with immunomodulatory and anti-inflammatory effects, and help degrade antinutritional factors, improving bioavailability [4,27,28,29,30]. These microbes can transiently colonize the gut, modulating microbiota composition and host immunity [31]. Table 2 summarizes the health benefits associated with some major fermented food category in India.

Fermentation significantly modifies the carbohydrate profile of Indian foods, leading to a reduction in their glycemic index (GI) (Table 3). LAB and yeasts metabolize starches and sugars into organic acids such as lactic, acetic, and propionic acids, which slow gastric emptying, inhibit α-amylase activity, and consequently reduce postprandial glucose levels [32].

Microbial fermentation promotes the formation of resistant starch, lowers the proportion of digestible carbohydrates, and enhances dietary fiber content [33]. Fermented staples such as idli, dosa, and dhokla show 20–30% lower GI compared to unfermented counterparts [34,35]. Traditional cereal-based products like ambali (fermented millet), pakhala, and pazhankanji (fermented rice) also demonstrate reduced glycemic load due to the presence of organic acids and LAB-mediated starch modification [36] (Table 3).

In addition, fermentation improves protein digestibility and increases bioactive peptide and soluble fiber content, contributing to delayed glucose absorption [37]. Regular intake of fermented foods supports gut microbial diversity, particularly lactobacilli and *Bifidobacterium* spp., enhancing short-chain fatty acid production and improving insulin sensitivity [38]. Overall, microbial fermentation transforms staple Indian foods into functional dietary components with measurable benefits for glycemic regulation and metabolic health.

## 7. Interlinking Microbial Diversity with Gut Microbiota

Consumption of fermented foods introduces live microbes and metabolites that modulate gut microbiota composition and function. Studies show that Indian diets rich in fermented foods support beneficial bacteria like lactobacilli, *Bifidobacterium*, and *Faecalibacterium*, which underpin metabolic and immune health [39]. Indian fermented foods exert a significant influence on gut microbiota composition and function, providing benefits through multiple mechanisms. These foods introduce live beneficial microbes like lactobacilli, *Bifidobacterium*, and *Weissella* species, which can transiently colonize the gut and interact with resident microbes. Their metabolites, especially short-chain fatty acids (SCFAs) such as butyrate and propionate, promote gut epithelial health, modulate inflammatory responses, and support immune homeostasis. Additionally, many LAB produce antimicrobial substances—bacteriocins, hydrogen peroxide, and reuterin—that protect against pathogens and enhance microbial ecosystem stability in the gut [5,31,40].

Empirical studies in Indian populations demonstrate that regular consumption of fermented foods (dahi, idli, kanji, fermented legumes) correlates with increased abundance of SCFA-producing bacteria such as *Faecalibacterium*, lactobacilli, and *Bifidobacterium*, which are hallmarks of a healthy and diverse gut microbiome. Certain fermented foods have also been linked to reduced incidence of metabolic and inflammatory conditions through modulation of gut microbiota composition [4,38,39,40,41,42].

Longitudinal observations indicate seasonal and regional shifts in gut taxa mediated by diet and fermented food intake, with taxa like *Prevotella* enriched in traditional diets rich in fibrous fermented foods. Examples include widely consumed Indian fermented foods like dahi, which is more commonly consumed in summer due to its cooling properties, and hawaijar, a fermented soybean product produced through alkaline fermentation by *Bacillus*, which is more frequently consumed in winter with the traditional belief that it promotes winter wellness. These dynamic interactions underscore the potential for fermented foods to contribute to microbial resilience and functional plasticity in diverse Indian communities [13,43,44].

Randomized controlled trials (RCTs) on fermented milk products containing lactobacillli (e.g., *L. acidophilus* La-5, *L. paracasei* F19) and *Bifidobacterium* (e.g., *B. lactis* BB-12) show symptom relief in irritable bowel syndrome (IBS) patients. The consumption improved the quality of life, reduced bloating, and better gut motility over 8 weeks compared to controls, although some trials report appears to be trends rather than statistical significance [42]. A symbiotic fermented milk trial (NCT02391220) assessed IBS symptoms and quality of life, finding potential benefits from multi-strain probiotics in enhancing gut barrier function and lowering inflammation. In Indian children, dahi supplementation increased anti-inflammatory IL-10 cytokines and lymphocyte counts more than plain milk, supporting immune modulation.

Recent literature highlights long-term (>10 years) consumption of Indian fermented foods like dahi and hawaijar stabilizing Bacteroidota fluctuations seasonally, boosting SCFA levels (acetate, propionate, butyrate) but sometimes reducing overall microbiota diversity and load [43]. Studies on northeast Indian products (e.g., hamei, marcha) confirm LAB probiotics inhibit pathogens like *Listeria*, potentially lowering abortion rates via immunity enhancement, while rice-based ferments show probiotic potential for gut balance. Gaps persist in large-scale Indian RCTs for metabolic diseases, but evidence supports microbiota-targeted benefits for inflammation and IBS [26].

## 8. Global Market Position and Consumer Trends of Indian Fermented Foods

Indian fermented foods have transitioned from household traditions to a fast-growing segment within the global functional food economy, reflecting both cultural export and health-oriented consumer dynamics. The global fermented foods market is projected to expand from USD 259.3 billion in 2025 to USD 398.8 billion by 2034, driven by probiotic awareness, post-COVID wellness trends, and demand for plant-based fermentation products. Asia-Pacific leads this expansion, accounting for the largest market share due to cultural familiarity and retail availability of traditional ferments. In India, the fermented food and beverage sector was valued at USD 22.8 billion in 2024 and is forecasted to reach USD 41.9 billion by 2033, registering roughly 7 percent annual growth. The Indian market now merges tradition with innovation, ready-to-cook idli dosa batters, probiotic curds, and kombucha exemplify how heritage products are being reformulated for urban consumers. Brands like Tata Consumer Products and iD Fresh Foods have extended traditional fermented products into premium and functional beverage categories. The Food Safety and Standards Authority of India (FSSAI) supports this growth through fortification initiatives and quality standardization frameworks.

Indian fermented food exports currently represent 2 percent of global processed food exports, yet are rapidly increasing as products like pickles, kanji, idli batters, and yogurt derivatives gain traction across diaspora and health driven Western markets. Indian pickles and fermented condiments are being rebranded as artisanal superfoods in Europe and North America, while fermented rice and millet products are drawing interest for their low gluten and probiotic profiles.

Consumer trends and market drivers for Indian fermented foods reflect a dynamic convergence of health awareness, technology, and lifestyle shifts. Growing probiotic and gut health awareness serves as the primary catalyst, positioning Indian fermented foods within the global “microbiome nutrition” narrative. Technological modernization, particularly through AI-powered fermentation monitoring and sustainable upcycling, has enhanced scalability, ensured quality consistency, and enabled innovation in product development. Simultaneously, demand peaks are evident in urban retail and e-commerce channels, where functional fermented snacks and beverages resonate with global “clean label” and craft food trends, reflecting consumers’ increasing preference for natural, traceable, and health-oriented products.

Overall, Indian fermented foods have progressed from household staples to globally traded wellness commodities. While India remains a regional leader in innovation and tradition convergence, its influence on the global fermented food market is poised to expand sharply through cultural branding, probiotic research integration, and sustainable food manufacturing initiatives. Commercializing Indian fermented foods requires a combination of quality assurance, innovative packaging, and effective marketing. Producers should focus on modern fermentation techniques, hygienic manufacturing, and leveraging India’s rich culinary heritage to build consumer trust. Product differentiation through highlighting health benefits, labelling, and extending shelf life with advanced packaging helps appeal to health-conscious markets. Multi-channel distribution, consumer education, and collaboration with research and industry can further expand reach and credibility. With growing demand for probiotic and functional foods, these integrated strategies collectively could enable traditional Indian ferments to enter broader domestic and international markets successfully.

## 9. Limitations and Knowledge Gaps

Despite the rapidly expanding body of work on Indian fermented foods, most available studies remain predominantly observational, cross-sectional, or descriptive. They often focusing on cataloguing microbial taxa, documenting traditional practices, or reporting in vitro functionalities rather than establishing causality between specific microbes, metabolites, and clinically relevant outcomes in human populations. This bias limits the strength of inferences that can be drawn about long-term health benefits, disease risk modification, or dose–response relationships for regular consumers of these foods.

At the microbial resolution level, only a fraction of the documented diversity has been characterized at the strain level. Even fewer strains have undergone rigorous functional validation for probiotic traits, safety, and techno-functional performance in relevant food matrices. Many sequencing-based reports provide genus- or species-level profiles without subsequent isolation, genome-scale characterization, or in vivo confirmation of health effects; thus, creating a disconnect between omics-derived signatures and strain-based mechanisms suitable for use as defined starter cultures or next-generation probiotics.

There is also a scarcity of large-scale, well-controlled randomized controlled trials in Indian populations. Existing studies rarely test specific fermented foods or standardized indigenous starters against appropriate control products. Consequently, evidence for outcomes such as glycemic control, metabolic syndrome, immune modulation, and microbiome restoration remains limited. Existing intervention studies often involve small sample sizes, short durations, heterogeneous products, and composite endpoints, which constrain meta-analysis and hinder the development of evidence-based dietary recommendations or regulatory health claims for Indian ferments.

Another major gap lies in translating multi-omics data into validated, regulatorily acceptable health claims and product specifications. Although multi-omics approaches have revealed candidate pathways and bioactive molecules, their translational application remains limited. Few studies have systematically progressed these insights through pilot-scale fermentation, in vitro digestion, and mechanistic host models. As a result, reproducible benefits supported by human trials and associated with well-defined microbial consortia or biomarkers are still scarce.

Finally, substantial variability and safety concerns remain around the widespread use of undefined household starter cultures and spontaneous backslopping in non-industrial settings. This is especially relevant for rice beers, soybean products, fermented fish, and spice-rich condiments. Batch-to-batch fluctuations in microbial composition can affect organoleptic quality and functional potency. They can also increase risks such as biogenic amine accumulation, mycotoxin contamination, and the carriage of transmissible antimicrobial resistance determinants [44]. Altogether, these concerns underscore the need for regionally grounded yet standardized indigenous starter systems, minimal process control metrics, and risk-based surveillance frameworks as these products transition to commercial scales.

## 10. Conclusions

The global future of Indian fermented foods appears highly promising, driven by rising consumer interest in gut health, plant-based nutrition, and functional foods. India’s extraordinary microbial and cultural diversity has given rise to a wide spectrum of fermented products, from cereal legume batters and pickled vegetables to dairy, fish, and bamboo shoot ferments; each harbouring region-specific beneficial microbiota. With the global fermented food market projected to expand steadily in the coming decade, Indian ferments hold significant potential for international recognition as sustainable, microbiome friendly, and culturally distinctive foods. Compared to other Asian fermented foods (for example, Japanese miso/natto or Korean kimchi), Indian traditional fermented cuisines are distinguished by their breadth of substrates (cereal and pulse batters, dairy, meat/fish, bamboo shoots, rice beers, floral liquors) and strong regional partitioning of microbial guilds across agroecological zones. While East Asian ferments often rely on relatively well-standardized koji or kimchi starter consortia, many Indian products still depend on semi-structured indigenous starters (marcha, hamei, ranu, herbal/leaf wraps), yielding higher microdiversity but greater batch-to-batch variability and less codified strain banks for industrial use. Technologically, Korea and Japan have already translated traditional microbiomes into defined commercial starter systems with robust quality assurance, whereas Indian ferments remain underexploited despite comparable or greater ecological richness and documented benefits for glycemic control and gut microbiota modulation.

Advanced multi-omics approaches integrating genomics, transcriptomics, proteomics, and metabolomics now offer powerful tools to decode microbial strain level diversity and metabolic traits, paving the way for personalized nutrition aligned with individual genetics and dietary habits. Indigenous starter cultures derived from native microbes can enhance product safety, sensory attributes, and health benefits while preserving ethno-microbial heritage and enabling standardized, large-scale production [6,11]. While the modern technological advancements continue to abridge the commercialization of traditional Indian fermented foods, several gaps still limit translation of indigenous starters into modern production. First, strain-level repositories and IP frameworks for Indian starter microbes lag behind those for Japanese koji or commercial kimchi cultures, resulting in poor valorization of region-specific taxa and risks of biopiracy. Second, process control and online monitoring for small and medium enterprises are underdeveloped; high-throughput qPCR, targeted amplicon sequencing, and low-cost biosensors used in cheese and kimchi production could be adapted to track keystone taxa and functional genes during Indian batter, vegetable, and rice-beer fermentations. Third, there is a disconnect between omics insights and pilot-scale validation: few studies systematically test omics-derived consortia in controlled fermenters, measure techno-functional traits (acidification kinetics, rheology, flavor volatiles), and couple these with in vitro digestion or human microbiome readouts. Finally, regulatory and labelling frameworks for “indigenous starter–based functional foods” remain nascent, which slows the emergence of GI-style, terroir-labelled Indian ferments with clearly articulated microbial and health value propositions [42].

Indian fermented foods position strongly in the health-conscious market as authentic, low-GI, microbiome-supporting staples with clinical evidence for glycemic control and metabolic health, outpacing many Western functional foods in cultural resonance and substrate diversity. Clinical data showing 20–30% GI reduction in idli/dosa/pakhala via organic acids and resistant starch, plus SCFA-mediated insulin sensitization, support explicit low-GI and blood sugar management claims. This appeals highly to prediabetic/T2DM consumers (global market > 500 M people) [43]. Trials with dahi and fermented cereals demonstrate HbA1c reductions (0.3–0.8%) and improved lipid profiles, rivaling pharmaceutical adjuncts but with superior safety and cultural familiarity. SCFAs from pazhankanji, ambali, and kinema boost butyrate-producing taxa (lactobacilli, *Faecalibacterium*), enhancing gut barrier and immunity—key differentiators vs. generic probiotics, with Indian diets linked to resilient microbiomes in cohort studies. “Probiotic heritage foods” branding leverages transient colonization and metabolite effects, targeting the $60 B global gut health market growing 8% annually. Thus, clinical studies involving diverse populations are imperative to establish mechanistic links between fermented food microbiota and host physiology, particularly in chronic disease prevention, immune modulation, and microbiome restoration. Cross-disciplinary collaborations among microbiologists, nutritionists, food technologists, and public health experts will be essential to translate these findings into functional foods, dietary guidelines, and therapeutic interventions tailored to India’s cultural and microbial diversity [40,44,45,46].

Safety assessment of Indian fermented foods needs to balance the benefits of rich beneficial, indigenous microbiota with documented risks from biogenic amines (BA), mycotoxins, and antibiotic resistance genes, particularly in products relying on the undefined, household cultures. Biogenic amines (such as histamine, tyramine, putrescine, cadaverine) are of concern in high-protein Indian ferments such as fermented fish (e.g., ngari-type products), soybean foods (kinema, hawaijar), and some dairy, where decarboxylase-positive LAB or contaminant Enterobacteriaceae can accumulate BA under suboptimal salt, temperature, and hygiene conditions. This often mirror the global patterns seen in other traditional fish and meat products [44,47]. Current evidence emphasizes that BA levels can provoke adverse reactions even below classic “toxicity” thresholds in sensitive consumers, further underscoring the need to select BA-negative or BA-degrading LAB and to implement process standardization when upscaling indigenous Indian ferments. In addition, mycotoxin risk arises where molds and mold-prone substrates intersect. For example, in rice-based amylolytic starters (marcha, hamei, ranu), cereal–pulse batters, millets, and spice-rich condiments can all be affected if raw grains or spices carry toxigenic *Aspergillus* or *Fusarium* strains, with aflatoxins and other mycotoxins persisting through fermentation due to their heat stability. Consequently, modernization agendas for Indian fermented foods should include raw material screening, preferential use of non-toxigenic mold strains (or LAB–yeast–only consortia where culturally acceptable), and adoption of rapid mycotoxin testing aligned with FSSAI and export-market limits. In parallel, emerging data show that LAB and co-occurring bacteria from traditional Indian ferments can harbor transmissible antibiotic resistance genes (e.g., tetracycline or macrolide determinants). Surveys in tribal hill-region foods have detected resistant staphylococci and coliforms alongside probiotic candidates, linking hygiene lapses and uncharacterized microbiota to potential AMR dissemination through the food chain [45,46,47]. This emphasizes the importance and need to transition from spontaneous, undefined backslopped cultures to “defined indigenous consortia” for the commercialization of the Indian fermented foods [48,49]. In fact, high-throughput qPCR, metagenomics, and chemometric monitoring can be used to set safety specifications, like BA thresholds, absence of specific mycotoxin genes, and mobile antimicrobial resistance gene markers to ensure the safe and sustained growth of this industry.

In conclusion, India’s diverse fermentation heritage with over 200 documented products, positions it as a leader in microbiome research, fostering international collaborations for functional foods and therapeutics. Indian fermented foods represent a scientifically rich and culturally grounded platform for gut microbiome modulation. As traditional knowledge converges with cutting edge microbial science, these foods have the potential to drive innovations in individualized nutrition, chronic disease prevention, and microbiome-based therapeutics, firmly positioning India’s fermentation heritage within the global health and food landscape. This integrates traditional wisdom with precision nutrition, addressing global challenges like antibiotic resistance through natural antimicrobials.

## 11. Future Perspective

Future perspectives for Indian fermented foods emphasize the development of defined indigenous starter cultures derived from region-specific sources. Multi-omics approaches can be used to build curated strain banks that are free of biogenic amines, mycotoxins, and antimicrobial resistance genes, enabling safe and standardized production.

Pilot-scale validation of these omics-informed microbial consortia in controlled fermenters is essential. Such studies should integrate real-time process monitoring with in vitro digestion models to confirm techno-functional traits and health-related benefits, including low glycemic index effects and short-chain fatty acid production.

Integrating fermentation science with personalized nutrition will require adaptive clinical trials. These trials should link specific microbial consortia to host genotypes, baseline mgicrobiomes, and metabolic phenotypes, particularly for targeted applications in prediabetes and type 2 diabetes mellitus.

Finally, the establishment of FSSAI aligned regulatory frameworks for functional and probiotic claims is critical. Clear safety specifications and terroir-based designations will facilitate the responsible commercialization of these microbiome-friendly heritage foods.

## Figures and Tables

**Table 1:** Overview of Indian fermented foods. Based primarily on ref [13,14].

Types of Fermented Food	Primary Substrate	Dominant Microbial Groups	Functional Properties	Geographical/Ethnic Association
Fermented Cereals	Rice, Lentils	Lactobacilli, *Weissella*, *Leuconostoc*	Lactic acid production, texture leavening, vitamin synthesis	South India (Idli, Dosa)
Legume/Pulse Fermentations	Soybean, Black gram	*Bacillus subtilis*, *Bacillus* spp.	Alkaline fermentation, protein hydrolysis, flavour development	Northeast/Eastern India (Kinema, Basi)
Fermented Dairy	Milk	Lactobacilli, *Streptococcus thermophilus*, *Enterococcus*	Probiotic traits, bioactive peptides, immune modulation	All India (Dahi, Lassi)
Fermented Vegetables	Leafy vegetables, bamboo shoots	Lactic acid bacteria, Yeasts, Fungi	Antioxidant retention, acidification, flavour enhancement	Northeast, Himalayas
Fermented Meat and Fish	Fish, Meat	Halophilic bacteria, Lactic acid bacteria	Protein degradation, flavour development	Coastal and Tribal Regions

**Table 2:** Suggested Health Benefits Associated with Selected Indian Fermented Foods [28,29].

Fermented Food	Originated from	Suggested Health Benefits
Curd (Dahi)	Pan India	Enhances digestion, improves immunity, provides probiotic support
Idli & Dosa	Tamil Nadu	Facilitates nutrient absorption, eases digestion
Kanji	Punjab	Acts as gut cleanser, antioxidant, supports digestive balance
Shrikhand	Maharashtra	Provides beneficial probiotics, supports digestive and metabolic health
Chaas (Buttermilk)	North India	Cooling, aids in digestion, improves metabolism
Pakhala	Odisha	Light and hydrating, promotes gut motility, reduces bloating
Appam	Kerala	Easily digestible, provides energy and probiotic benefits
Kinema	Sikkim	High in protein, enhances digestibility, supports gut health
Gundruk	Sikkim	Rich in natural probiotics, supports robust gut microbiota
Apong	Assam/Nagaland	Stimulates energy levels, probiotic-rich, part of traditional diets
Axone (Akhuni)	Assam	High in protein, anti-inflammatory, offers distinct flavour profiles
Ngari	Manipur	Rich in proteins and omega-3 fatty acids, supports overall nutrition
Mahua	Madhya Pradesh	Traditional alcoholic beverage with energy-boosting and cultural benefits
Selroti	Sikkim	Nutritious, easily digestible, supports balanced energy levels
Chhurpi	Sikkim/Himalayan	High in protein, beneficial for bone and dental health, probiotic source
Chhang	Eastern Himalayas	Provides energy and sustenance, supports a healthy gut microbiome
Dhokla	Gujarat	High in Plant-based protein
Hawaijar	Manipur	Anti-inflammatory properties
Khorisa	Assam	Packed with fiber and vitamin
Handia	Jharkhand	Vitamin B-complex, essential for metabolism
Chak Hao Kheer	Manipur	Support health
Sol Kadhi	Goa	Helps in blood purifier
Jolada Ambli	Karnataka	Low-calorific

**Table 3:** Glycemic effects of some Indian fermented foods.

Food	Primary Substrate	Glycemic Effect
Idli/Dosa	Rice + urad dal	Reduced GI (20–30%), Increased resistant starch
Dhokla	Bengal gram + rice	Reduced GI, Increased soluble fiber
Ambali	Finger millet	Low GI, probiotic effect
Pazhankanji/Pakhala	Rice	Reduced available carbohydrate
Kambu koozh	Pearl millet	Low GI, Increased SCFA production

## Data Availability

No new data were created or analyzed in this study.
